# Wilms’ tumour suppressor gene 1 (WT1) is involved in the carcinogenesis of Lung cancer through interaction with PI3K/Akt pathway

**DOI:** 10.1186/1475-2867-13-114

**Published:** 2013-11-14

**Authors:** Xi Wang, Ping Gao, Fang Lin, Min Long, Yuanyuan Weng, Yongri Ouyang, Li Liu, Junxia Wei, Xi Chen, Ting He, Huizhong Zhang, Ke Dong

**Affiliations:** 1 Department of Clinical Diagnosis, Tangdu Hospital, Fourth Military Medical University, Xi’an, China; 2 Department of Gynecology and Obstetrics, Tangdu Hospital, Fourth Military Medical University, Xi’an, China

**Keywords:** WT1, Lung cancer, PI3K/AKT

## Abstract

**Absract:**

Although studies have shown the oncogene WT1 is overexpressed in lung cancer, there is no data showing the implication of WT1 in lung cancer biology. In the present study, we first demonstrated that isotype C of WT1 was conservely overexpressed in 20 lung cancer patient specimens. Knockdown of WT1 by small interference RNA (siRNA) transfection resulted in a significant inhibition of cell proliferation, induction of cell cycle arrest at G_1_ phase, and the expression change of BCL-2 family genes in WT1^+^ A549 cells. Furthermore, we found that DDP treatment could decrease the WT1 mRNA expression level by 5% and 15% at a dose of 1 μg/ml, by 25% and 40% at a dose of 2 μg/ml for 24 and 48 h, respectively. In the mean time, DDP treatment also reduced the PI3K/AKT pathway activity. Further analysis by using siRNA targeting the AKT-1 and the PI3K pathway inhibitor Ly294002 revealed that the AKT-1 siRNA reduced the WT1 expression effectively in A549 cells, and the same result was observed in Ly294002 treated cells, indicating that DDP treatment could down regulate WT1 expression through the PI3K/AKT pathway. Of particular interest, knockdown of WT1 also inhibited the AKT expression effectively, Chip assay further confirmed that WT1 is a transcription factor of AKT-1. We thus concluded that there is a positive feedback loop between WT1 and AKT-1. Taken together, DDP treatment downregulates the WT1 expression through the PI3K/AKT signaling pathway, and there is a feedback between WT1 and AKT-1; WT1 is involved in cellular proliferation in A549 cells, WT1 inhibition in combination with DDP will provide a new light for lung cancer therapy.

## Introduction

Lung cancer is one of the most common cancer worldwide, and is the leading cause of cancer related death [1,2]. *cis*-Diamminedichloroplatinum (II) (cisplatin, DDP) is one of the most effective drugs currently available for the treatment of lung cancer [3]. Although advances in therapy for lung cancer have been achieved by combination chemotherapy with cisplatin or carboplatin plus etoposide [4], with the addition of radiation therapy in limited stage, and the overall patients’ outcome has been substantially improved, the majority of patients with limited stage suffer relapse after concurrent chemoradiotherapy [5]. Therefore, new effective therapeutic strategies for lung cancer are urgently needed, and the molecular mechanisms are needed to be demonstrated.

The Zinc finger protein WT1 was initially identified as a tumor suppressor gene in Wilms’ tumors [6]. It is a modular transcription factor with an NH_2_-terminal glutamine and proline-rich domain involved in self-association, transcriptional repression, and transcriptional activation [7]. The four zinc finger structure in the COOH terminus of WT1 is involved in DNA and RNA binding, nuclear localization, and protein-protein interactions. WT1 encodes for 10 exons and generates various mRNA species. Through alternative splicing, there are four predominant protein isoforms of WT1 that differ by the presence of a 17-amino-acid of exon 5 and a 3-amino-acid insert (lysine, threonine, and serine: KTS) that is found at the 3′end of exon 9 [8,9]. The different isoforms are referred to as A, B, C, and D, where A lacks both the 17-amino-acid and KTS inserts; B contains the 17-amino-acid insert but lacks KTS; C lacks the 17-amino-acid insert but contains KTS; and D contains both the 17-amino-acid and KTS inserts [10].

Despite WT1 originally recognized as a tumor suppressor, a growing body of experimental evidences indicates that WT1 has oncogenic function in leukemias and a variety of solid tumors e.g. colon cancer, head and neck squamous cell cancer (HNSCC), pancreatic cancer, salivary gland cancer [11], ovarian cancer [12-14], and lung cancer [15,16]. So WT1 is a universal tumor antigen and consequently a good therapeutic target for the development of gene therapy strategies. Recently, WT1 was ranked first in a list of 75 cancer antigens [17].

The expression of WT1 in lung cancer has prognostic effects, Oji et al. [18] found that high level of WT1 IgG antibody expression in lung cancer is associated with a worse prognosis. Studies also shown that WT1 is an effective immunotherapeutic target [19]. A report showing WT1 was overexpressed in 54/56 (96%) de novo non-small cell lung cancers (NSCLCs) and 5/6 (83%) de novo small cell lung cancers (SCLCs) specimens [15]. Although in this report, the authors observed a correlation between WT1 expression and patient survival, there is no data showing the implication of WT1 in lung cancer biology. In the present study, we found that WT1 is also over expressed in tumor specimens in a high proportion of patients with lung cancer, use directed sequencing we also found that isoform C type of WT1 was conservely expressed in lung cancer barely without mutation. We also found that knockdown of WT1 in lung cancer cells induces the cell cycle arrested at the G_1_ phase, reduces the expression of antiapoptotic genes, whereas this maneuver enhances the expression of proapoptotic genes. Moreover, we showed that treatment of DDP, the standard chemotherapy reagent of lung cancer, reduced the WT1 expression in lung cancer cells through inhibition of the PI3K/AKT signaling pathway, furthermore, WT1 is a transcriptional factor of AKT, and there is a positive feedback loop between WT1 and AKT expression. We also pointed out that inhibition of WT1 expression increased the sensitivity of the cells to chemotherapeutic compound DDP, and enhanced DDP induced apoptosis. These data implicate the involvement of WT1 isotype C in lung cancer progression and resistance to chemotherapy, WT1 inhibition in combination with DDP treatment will provide a new light for novel lung cancer therapy.

## Materials and methods

### Tissue samples

Lung cancer tissues and normal-appearing lung tissues were obtained from patients with lung cancer at Tangdu Hospital, with informed consent. Clinicopathologic features in lung cancers are listed in Table 1. NSCLC stages were classified according to the UICC TNM classification.

**Table 1:** Clinical features of patients with lung cancer and WT1 expression in lung cancer tissues

**Patient**	**Age (year)**	**Sex**	**Histology**	**TNM**	**Stage**	**WT1 mRNA level**
1	69	F	Ad	T1N1M0	IIA	8.3×10^-1^
2	55	M	Ad	T1N1M0	IIA	2.8×10^0^
3	56	F	Ad	T2N1M0	IIB	0.7×10^0^
4	50	M	Ad	T2N0M0	IB	9.3×10^-1^
5	63	F	Ad	T2N1M0	IIB	0.9×10^0^
6	63	M	Sq	T1N0M0	IA	0.8×10^-2^
7	56	M	Sq	T1N0M0	IA	3.8×10^-1^
8	64	F	Sq	T2N1M0	IIB	3.4×10^0^
9	47	M	Sq	T1N0M0	IA	0.5×10^-1^
10	56	M	Sq	T1N0M0	IA	4.5×10^-1^
11	63	M	Sq	T2N1M0	IIB	2.1×10^0^
12	64	M	Sq	T1N0M0	IA	0.3×10^-2^
13	54	M	Sq	T2N1M0	IIB	3.7×10^0^
14	71	M	Sq	T1N2M0	IIIA	5.4×10^0^
15	61	M	Sq	T1N1M0	IIA	1.4×10^0^
16	75	M	Sq	T2N1M0	IIB	3.5×10^0^
17	53	M	Sq	T2N0M0	IB	0.2×10^-1^
18	59	M	Sq	T1N2M0	IIIA	3.1×10^0^
19	76	M	Sq	T2N0M0	IB	0.5×10^0^
20	66	M	Sq	T2N0M0	IB	7.3×10^-1^

Lung cancer tissues were obtained from patients with lung cancer at Tangdu Hospital, with informed consent. NSCLC stages were classified according to the UICC TNM classification and Clinicopathologic features of each lung cancers are listed. WT1 expression levels in NSCLCs were examined by means of quantitative real-time RT-PCR and listed in the last column.

### Patients and cell lines

The WT1^+^ A549 and WT1^-^ PC14 non-small-cell lung cancer cell lines were obtained from ATCC (Rockville, Mass., USA) and propagated in the recommended media. Lung cancer specimens were obtained from the primary tumor site during operation (Thoracic Surgical Departments of the Tangdu Hospital, Xi’an, China). For the use of these clinical materials for research purpose, prior patients’ consents and approval from the Institutional Research Ethics Committee were obtained. Under sterile conditions, tumor samples of 0.5 cm in diameter were taken and shock-frozen in liquid nitrogen. Matched normal tissue was taken in parallel for each patient and samples were evaluated by a pathologist immediately following dissection. Samples from macroscopically tumor-free margins of the operative specimens were also processed accordingly.

### Drugs and antibodies

DDP (min. 94%) was purchased from Sigma-Aldrich. A 10 mM DDP stock solution was dissolved in PBS and stored at −20°C until use. Ly294002 was purchased fron cell signaling, and A 10 mM Ly294002 stock solution was dissolved in DMSO and stored at −20°C until use. Monoclonal anti-human WT1 (F-6), and goat anti-mouse secondary antibody for Western blot were obtained from Santa Cruz Biotechnology, Inc. The antibody anti-pAKTser473 was obtained from Cell Signaling.

### Quantitative real-time RT-PCR

Quantitative real-time RT-PCR was performed using SYBR green master mix (Takara, Japan), according to the following PCR conditions: initial denaturation at 95°C for 3 min followed by 30 cycles of amplification at 95°C for 10 s and 60°C for 15 s. The amplified fluorescent signal was detected by Roche LightCycler 480 (Roche Diagnostics). Relative quantification was assessed using secondary derivative maximum (Roche Diagnostics). Gene expression was normalised to GAPDH and differences in expression measured relative to the control (A549 cells). For each sample, all experiments were repeated in triplicate using two independent cDNA extractions with RNA isolated from three independent RNA extractions.

### RT-PCR

Total RNA was prepared from 10^6^ A549 cells at the exponential growth phase of the cell lines using the RNAzol extraction protocol (Takara). Frozen tumor samples were put in RNAzol solution and disrupted in a 1-ml tissue homogenizer. Total RNA was extracted as previously described. Reverse transcription was performed, using SuperScript RNase H reverse transcriptase (Gibco BRL, Gaithersburg, Md., USA). Two pairs of primers were used to amplify the full length of the WT1 gene (1540 bp). The primer sequences for the 1-750 bp of the WT1 gene were 5′-atgctgcaggacccggcttcc-3′, 5′-ggatcctcatgcttgaatgagtggtt-3′; the primers for 751-1540 bp of the WT1 gene were 5′-ggatcccatgggccagcagggct-3′, 5′-tcaaagcgccagctggagtttgg-3′. A total of 30 cycles of the RT-PCR protocol were performed, and all of the PCR product were ligation with PMD18T vector, and sequenced by AUCT company (Beijing, China).

### siRNA transfection

siRNA was synthesised by genepharma, Shanghai, China. siRNA sequence targeting WT1 was synthesised. GGACUGUGAACGAAGGUUU corresponds to positions 28–46 within exon 8 of the WT1 open reading frame, according to Glienke’S article [20]. Cells were seeded into six-well plates at a density of 5 × 10^5^ cells per well in 6 well plates or 1 × 10^4^ cells per well in 96 well plates 24 h before transfection. A549 and PC14 cells were transfected by using the lipofectamine^2000^ transfection reagent (invitrogen) following the manufacturer’s instructions. For each transfection, 10 pmol of precursor WT1 siRNA or negative control siRNA in 250 μl of Opti-MEM (Invitrogen) was respectively mixed with 250 μl of Opti-MEM that contained 5 μl of lipofectamine^2000^ reagent which had been pre-incubated for 5 min at room temperature. The mixture was incubated for 20 min at room temperature and then added to the cells. The cells were harvested 24 h after transfection for flow cytometry, RT-PCR, and Western blot analysis. In some experiments, 24 h post transfection, the cells were incubated with different concentrations of DDP for cell proliferation assay. All transfections were performed in triplicate for each time points.

### Construction of WT1 expression vector

WT1 cDNA was amplified by PCR method using A549 cDNA as template. The following primers were synthesized with KpnI and EcoRI restriction enzyme sites introduced: WT1 sense (5′-ggggtaccgccaccatgctgcaggacccggcttcc-3′) and antisense (5′-ccggaattctcaaagcgccagctggagtttgg-3′). The PCR products were then inserted into the KpnI and EcoRI sites of pcDNA3.1 vector and named pcDNA3.1-WT1.

### Plasmids transfection

293 T cells were seeded in 6-well plates cultured overnight to about 80% confluence, and then transfected with pcDNA3.1-WT1 or pcDNA3.1(control plasmid) using lipofectamine^2000^ transfection reagent following the manufacturer’s specifications, respectively. The cells were harvested 48 h after transfection for Western blot analysis.

### 3-(4, 5-Dimethyl-thiazol-2-yl)-2, 5-Diphenyltetrazolium bromide (MTT) assay

Cell proliferation was determined by the MTT (Sigma, ST. Louis, MO, USA) assay as described elsewhere [21] Briefly, the cells were plated in 96-well tissue culture plates at a density of 1 × 10^4^ cells per well and allowed to attach overnight, cells were transfected with si-WT1 for 24 h, and then treated with different concentrations of DDP for 48 h, then incubated with MTT (20 μl of 5 mg/mL) for 4 hours. The formazan precipitate was dissolved in 150 μl of dimethylsulfoxide (DMSO) and the absorbance at 570 nm was measured by a benchmark microplate reader (Bio-Rad, Hercules, CA).

### Cell cycle and apoptosis analysis

The effect of WT1 on cell cycle distribution was determined by flow cytometry using the cell cycle detection kit (Keygen, Nanjing, China). Briefly, 24 h post transfection, adherent cells were collected, washed twice with phosphate-buffered saline (PBS), then resupended in 0.2 mL PBS, then added the solutions according to the manufactories protocol. Before analysis, cells were resupended and then analyzed by flow cytometry. The relative proportions of cells in the G_1_, S and G_2_/M phases of the cell cycle were determined by the flow cytometry.

The apoptosis was also detected by flow cytometric analysis using the AnnexinV-PI Staining Kit (Roche Applied Biosciences, Mannheim, Germany). A549 cells were transfected with si-WT1 for 24 h, and then treated with or without 1 μg/ml of DDP for 24 h, cells were then washed in cold PBS and resuspended in binding buffer and added FITC-labeled AnnexinV and PI. After incubation for 10 minutes at room temperature, the cell suspentions were immediately analyzed using the flow cytometry. Three independent experiments were performed and the percentage of apoptotic cells was calculated with WinMDI software.

### Western blotting

Protein extract was electrophoresed on a 10% SDS-polyacrylamide gel, transferred to NC membrane. After blocking the membrane was incubated overnight at 4°C with primary antibodies. Primary antibodies were removed and the blots were extensively washed with TBST for three times, and then incubated for 1 h at room temperature with the secondary antibody. Following removal of the secondary antibody, blots were washed, and the specific bands were detected by enhanced chemiluminescence kit (Santa Cruz).

### Chromatin immunoprecipitation (ChIP) assay

1% formaldehyde cross-linked cells were sonicated, and centrifugated. The supernatants were incubated with WT1 antibody (C-19), IgG or no antibody as control at 4°C overnight with rotation. The immune complexes were precipitated with salmon sperm DNA/protein A-agarose (Santa Cruz) for 2 h at 4°C. DNA was recovered by extraction in phenol/chloroform/isoamyl alcohol followed by ethanol precipitation. The presence of AKT-1 promoter in the immune precipitates was confirmed by PCR, and the sequences of primers for the AKT-1 promoter following Chip assay were as follows: 5′-GGGATGAATAATGTTCCATAA-3′ and 5′-AAGCTGTTGAGGCAATGT-3′. PCR was performed with a melting temperature of 94°C for 15 seconds, followed by 47°C for 30 seconds, and then 72°C for 45 seconds for 40 cycles.

### Statistical analysis

All experiments were performed a minimum of three times. Data represent the mean ± SD calculated from multiple independent experiments. Statistically significant differences for data points represent P < 0.05 and were calculated by using either the unpaired t test. Quantitative real-time reverse transcription-PCR data were calculated with JMP 5 for Windows software (SAS Institute, Inc., Cary, NC). Differences between groups were estimated using the Student’s t test.

## Results

### Over expression of WT1 in lung cancer patients

WT1 expression levels in NSCLCs and normal-appearing lung tissues were examined by means of quantitative real-time RT-PCR. As shown in Table 1, all (100%) of 5 adenocarcinomas expressed WT1 ranging from 8.3 × 10^-1^ to 2.8 × 10^0^ (WT1 expression level in A549 lung cancer cells was defined as 1.0), and 15 squamous cell lung cancer tissues expressed WT1 at levels ranging from 0.3 × 10^-2^ to 5.4 × 10^0^. However, WT1 expression was detected in 0/10 normal lung tissues of patients. Expression of the WT1 gene could not be examined by immunohistochemistry because of the difficulty in obtaining enough cancer tissue to perform this test. This result showed over expression of the WT1 gene in NSCLCs.

### No detection of mutations or deletions in WT1 transcripts

**Figure 1:**
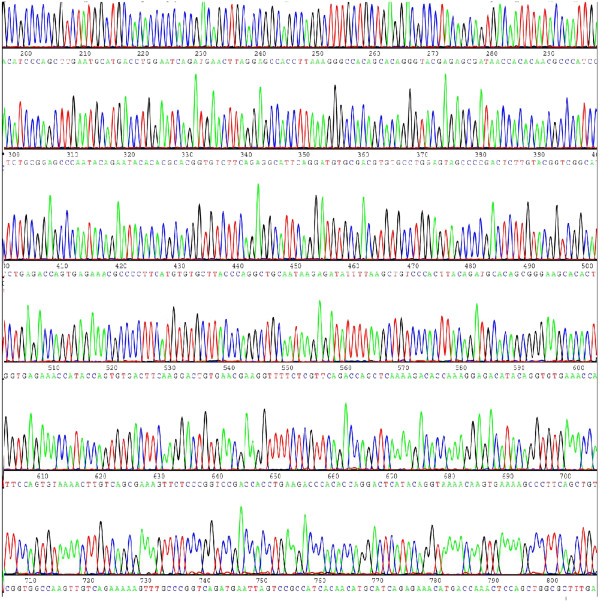
**One of the WT1 gene sequence from lung cancer specimen.** RNA was obtained from lung cancer samples, the RNA templates were then reverse transcribed into cDNA, and RT-PCR analysis was performed using the primers which covered sequences from the 3′ end of exon 1 to the whole of exon 10, to obtain the whole length sequence of WT1 gene. This figure shown part of the sequence result.

To determine whether WT1 transcripts expressed in lung cancer tissues and cancer cells have deletions and/or mutations, RT-PCR analysis was performed to obtain the whole length sequence of WT1 gene using the primers which covered sequences from the 3′ end of exon 1 to the 5′ end of exon 10. The sequencing result showed that in all the 22 samples, only 2 samples of lung cancer tissue express the isoform A of WT1 gene, others were stably expressing the isoform C of the WT1 gene, and no mutations and/or deletions were detected from exon 1 to exon 10, Figure 1 shows one of the sequencing result.

### siRNA targeting WT1 contributes to the cell growth inhibition and cell cycle arrest in WT1^+^ lung cancer cell line but not in WT1^-^ lung cancer cell line

**Figure 2:**
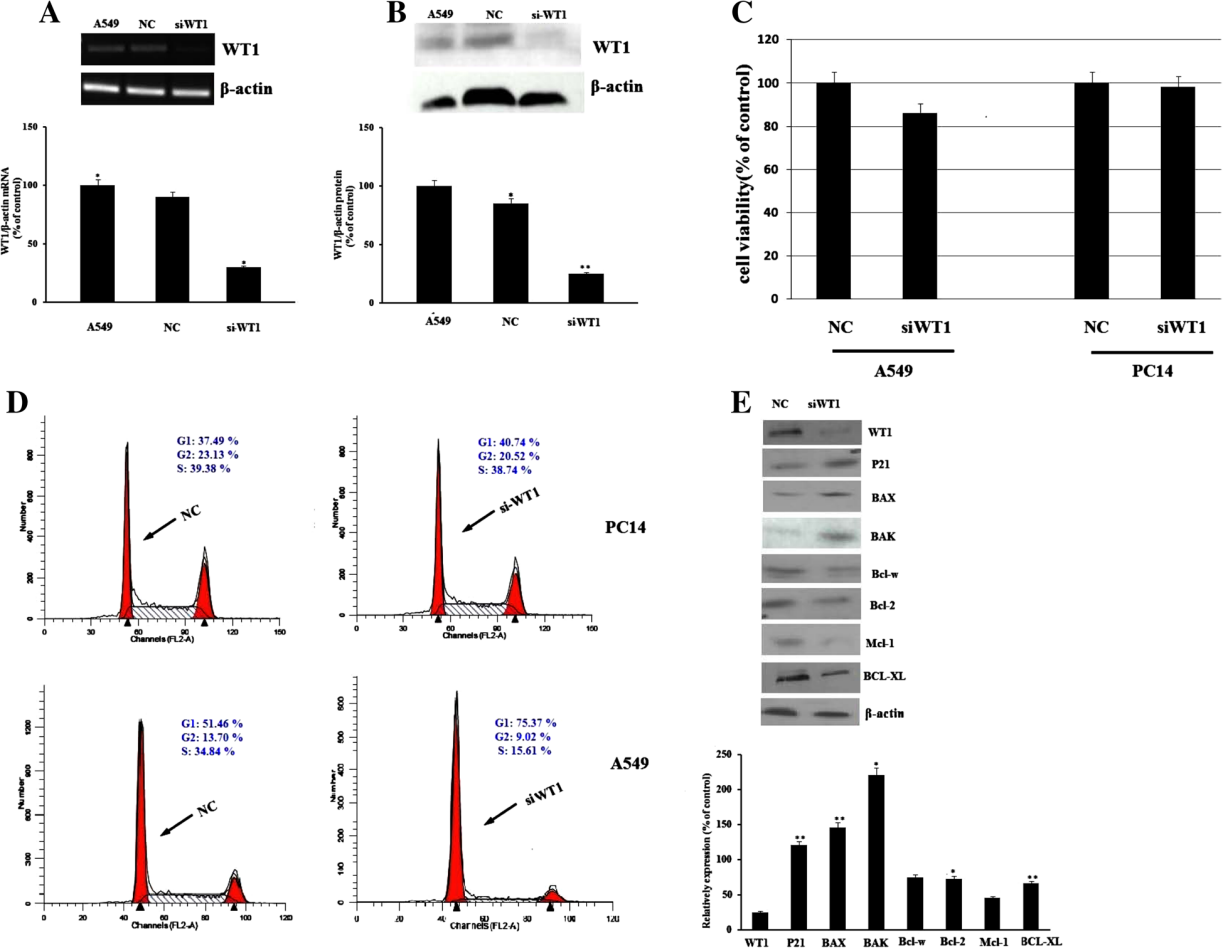
**Effect of WT1 siRNA transfection on the cell growth and cell cycle distribution of lung cancer cells, some pro-apoptotic or anti-apoptotic genes expression in A549 cancer cells. A**, **B**. A549 cells were transfected with the WT1 targeted siRNA for 24 h, RT-PCR and western blot were performed to detect the changes of the WT1 expression level. NC: Negative control siRNA; siWT1: siRNA target WT1. The P value shows the difference between siRNA transfected cells and parental cells; bars, SE; * = P < 0.05, ** = P < 0.01. **C**. A549 and PC14 lung cancer cells were transfected with WT1 siRNA or negative control siRNA, 72 h post transfection, MTT was performed to detect the cell proliferation. NC: Negative control siRNA; siWT1: siRNA target WT1. **D**. 24 h post transfection, the cell cycle profile was monitored by FACS analysis. The distribution of cells in the G_1_, S, and G_2_/M phases of the cell cycle were calculated and labeled. NC: Negative control siRNA; siWT1: siRNA target WT1. **E**. A549 cells were transfected with WT1 siRNA for 24 h, and then harvested and subjected to the Western blots for the pre-apoptotic genes of BAX, BAK, p21, anti-apoptotic genes Mcl-1, Bcl-xl, Bcl-W. NC: Negative control siRNA; siWT1: siRNA target WT1. The P value shows the difference between siRNA transfected cells and parental cells; bars, SE; * = P < 0.05, ** = P < 0.01.

We then designed experiment to evaluate the effect of WT1 knockdown on the proliferation of lung cancer cells. Figure 2A and 2B showed that transfection of the chosen siRNA reduced WT1 expression for nearly 65%. We then examined the effect of si-WT1 on proliferation of WT1^+^ A549 and WT1^-^ PC14 cell lines, cells were transfected with si-WT1 for 48 h and cell viability was then analyzed by MTT assay. The introduction of si-WT1 caused a remarkable inhibition of cell proliferation in A549 cells but not in PC14 cells compared with control siRNA transfected cells (Figure 2C).

The effects of si-WT1 on cell cycle progression were investigated using flow cytometry. A549 and PC14 lung cancer cells were transfected with si-WT1 for 24 h and then analyzed for cell cycle distribution by means of flow cytometry. si-WT1 increased the population of cells in the G_0_-G_1_ phase with a reduction of cells in the S phase in A549 cells but not in PC14 cells. Figure 2D showed in A549 cells, the G_1_ phase population was increased from 51.46% to 75.37%, and Figure 2E showed the cyclin-dependent kinase inhibitor1 p21/WAF1 was also increased by 50% post WT1 siRNA transfection, we thus hypothesize that the WT1 siRNA induces G_1_ phase arrest through the increase of p21. Data were represented as mean ± S.D. from at least three independent experiments, and indicated that si-WT1 led to cell proliferation inhibition and cell cycle arrest at the G_1_ phase in WT1^+^ A549 cells.

We also examined the expression change of Bcl-2 family members Bax, Bak, Bcl-w, Bcl-2, BCL-XL, and Mcl-1 post WT1 siRNA transfection. Figure 2E showed that the antiapoptotc genes BCL-XL and Mcl-1 expression was reduced by 33% and 55% respectively in WT1 siRNA transfected A549 cells. In contrast, the expression levels of pre-apoptotic proteins Bax and Bak were increased 1.46 fold and 2.2 fold. This was consistence with Tatsumi’s experiment which showed Bax was the downstream target of WT1 [22].

### Reduction of the WT1 expression in response to DDP in human lung cancer A549 Cells

**Figure 3:**
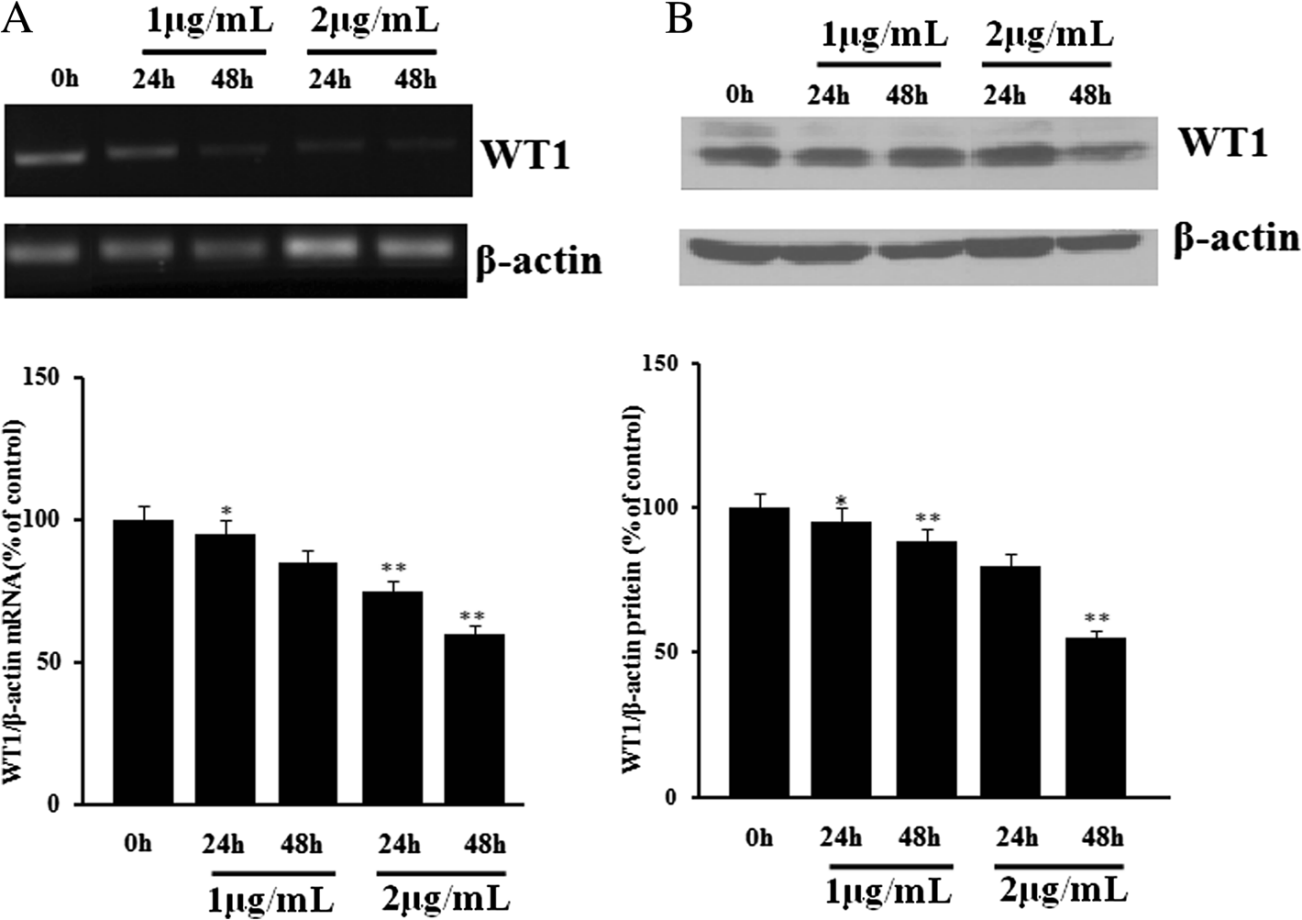
**Reduction of WT1 mRNA and protein expression post DDP treatment in A549 cells. A**. A549 cells were incubated in the present of 1 μg/ml or 2 μg/ml of DDP for different time points of 24 and 48 h, and the WT1 expression was detected by conventional RT-PCR method. The P value shows the difference between DDP treated cells and parental cells; bars, SE; * = P < 0.05, ** = P < 0.01. **B**. A549 cells were incubated in the present of 1 μg/ml or 2 μg/ml of DDP for different time points of 24 and 48 h, the WT1 expression was detected western blots. The P value shows the difference between DDP treated cells and parental cells; bars, SE; * = P < 0.05, ** = P < 0.01.

To determine the effect of the standard lung cancer chemotherapy reagent DDP on WT1 mRNA expression and protein level, A549 cells were incubated with DDP for 24 h and 48 h, respectively. The WT1 mRNA levels were decreased by 5% and 15% in response to 1 μg/ml DDP treatment, and by 25% and 40% in response to 2 μg/ml DDP treatment as determined with RT-PCR, the result of western blot assay was consistent with that of RT-PCR (Figure 3).

### DDP reduces WT1 expression via the PI3K/AKT signaling pathway

**Figure 4:**
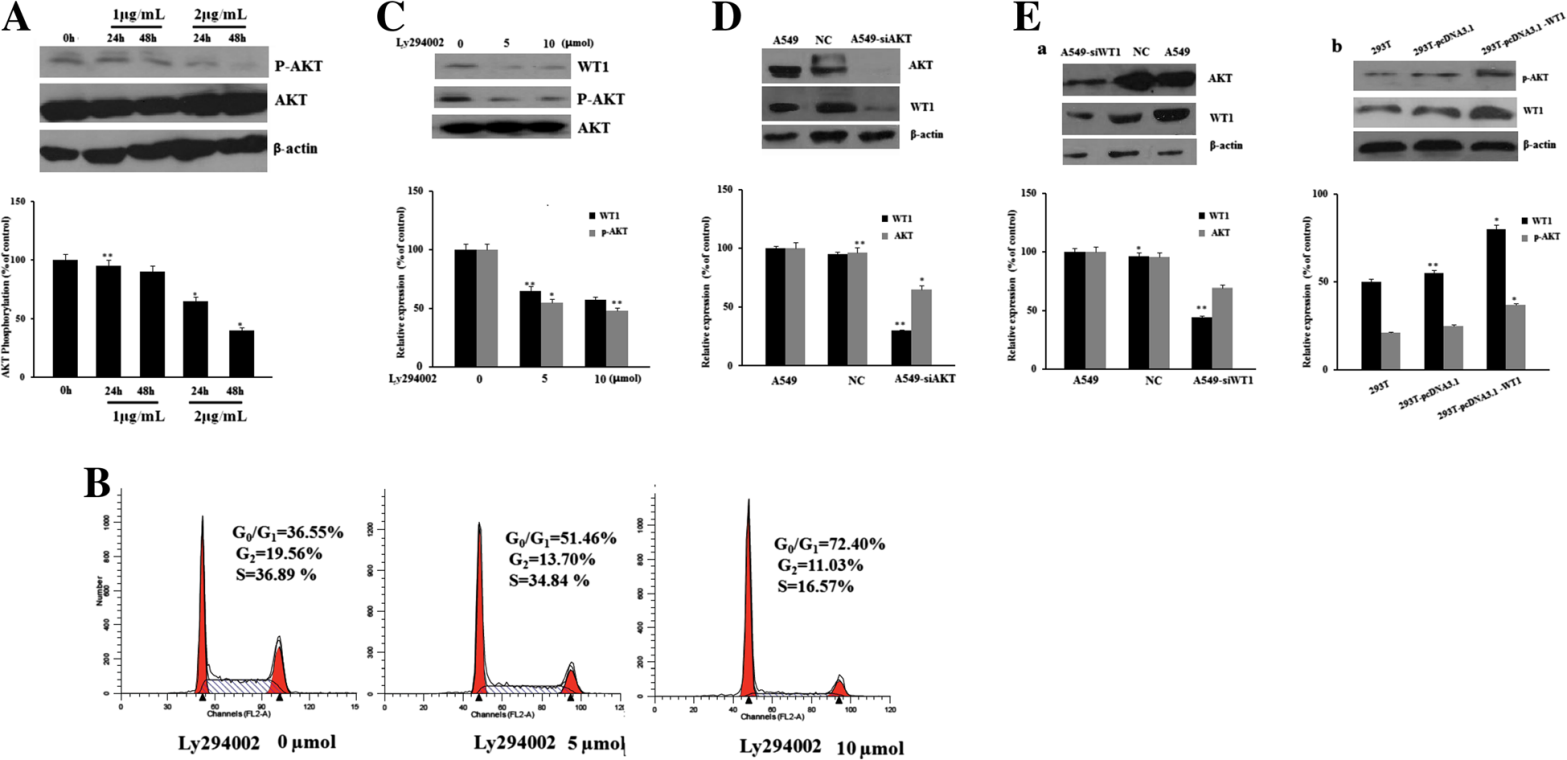
**The effect of DDP treatment on PI3K/AKT pathway activity. A**. A549 cells were incubated with 1 or 2 μg/ml of DDP for 24 h and 48 h, respectively, the total AKT and p-AKT was detected by western blot. The P value shows the difference between DDP treated cells and parental cells; bars, SE; * = P < 0.05, ** = P < 0.01. **B**. A549 cells were treated with 5 or 10 μmol of PI3K pathway inhibitor Ly294002 for 24 h, cells were then collected, cell cycle profile was monitored by FACS analysis. **C**. A549 cells were treated with 5 or 10 μmol Ly294002 for 24 h, cells were then collected, western blot methods were used to detect the WT1, p-AKT, and AKT expression. The P value shows the difference between Ly294002 treated cells and parental cells; bars, SE; * = P < 0.05, ** = P < 0.01. **D**. A549 cells were transfected with AKT-1 targeted siRNA for 24 h, cells were collected, western blot was used to detect the AKT and WT1 expression.NC: Negative control siRNA transfected cells; A549-siAKT: siRNA target AKT transfected cells. The P value shows the difference between siRNA transfected cells and parental cells; bars, SE; * = P < 0.05, ** = P < 0.01. **E**. a. A549 cells were transfected with WT1 targeted siRNA or control siRNA for 24 h, cells were collected, western blot was used to detect the p-AKT and WT1 expression. A549- siWT1: siRNA target WT1 transfected cells. b. 293T cells were transfected by pcDNA3.1-WT1 or control vector pcDNA3.1 for 24 h, cells were collected and then western blot assay was used to detect the expression level of p-AKT and WT1. 293T-pcDNA3.1: pcDNA3.1 transfected 293T cells; 293T-pcDNA3.1-WT1: pcDNA3.1-WT1 transfected 293T cells. The P value shows the difference between transfected cells and parental cells.

The oncogene AKT (or PKB) is involved in the regulation of cell survival, and the phosphatidylinositol 3-kinas (PI3K)/AKT signaling pathway regulates many normal cellular processes including cell proliferation, survival, growth and motility-processes, the aberrant activation of this pathway has been considered to be critical for tumorigenesis [23,24]. It has been demonstrated that over activation of PI3K/AKT pathway involved in the chemoresistance of tumor cell lines to DDP treatment [25,26]. Interestingly, we also found that DDP treatment reduced phospholated AKT but not total AKT expression (Figure 4A). Svensson et al. demonstrated that WT1 is the downstream target of AKT [27], we hypothesized that the DDP treatment might down regulate WT1 expression through the PI3K/AKT signaling pathway, so firstly we treated A549 cells with PI3K inhibitor (LY294002) for 24 h, as expected, inhibition of PI3K arrested the cell cycle at G1 phase (Figure 4B), and also resulted in a strong decrease of WT1 mRNA and protein expression (Figure 4C). These results making us conclude that WT1 mRNA expression is highly dependent on PI3K signaling. We then transfected A549 cells with siRNA targeting AKT, Figure 4D showed that AKT siRNA not only decreased the expression level of AKT but also reduced WT1 expression. To our surprise, the changes of WT1 expression also affect the AKT expression effectively. In WT1 siRNA transfected A549 cells, phosphorylated forms of AKT was reduced (Figure 4Ea). When we Transfected the 293 T cells with pcDNA3.1-WT1 which carrying the wild type WT1, we found that the phosphorylated form of AKT was increased (Figure 4Eb). These results indicated that DDP-induced downregulation of WT1 mRNA and protein is mediated via the PI3K/AKT pathway, and there is a positive feedback between AKT and WT1 expression.

### WT1 directly regulates AKT

**Figure 5:**
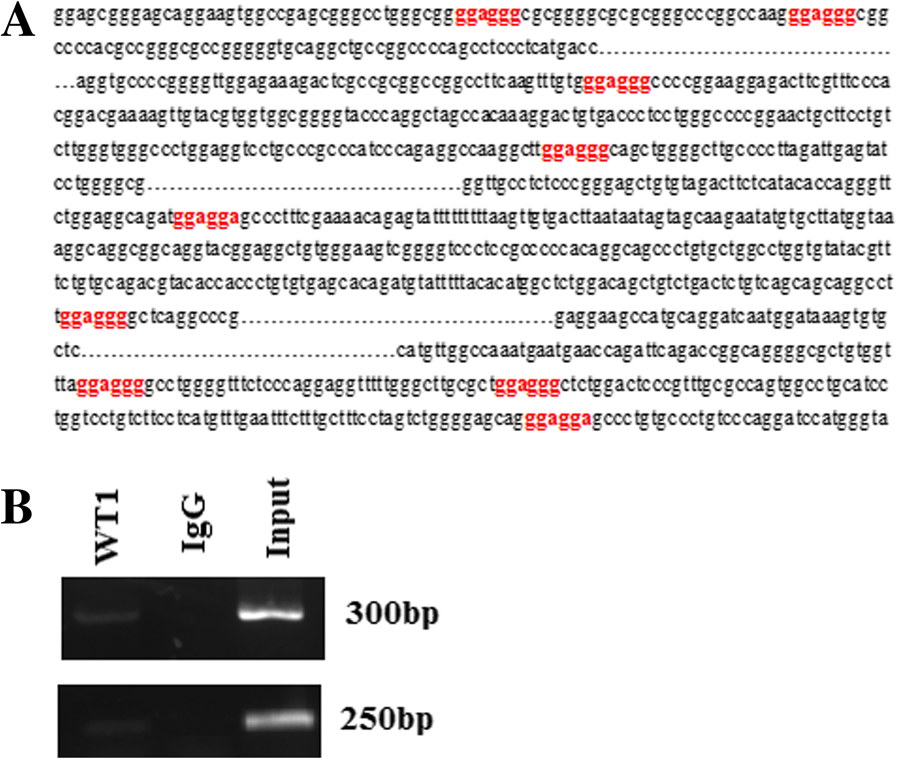
**WT1 regulates AKT expression through promoter binding. A**. The putative promoter region of AKT was predicted using the promoter scan website (http://www-bimas.cit.nih.gov/molbio/proscan). The WT1 binding motif in AKT promoter was predicted using the promoter and Transcription Factor Analysis web site (http://www.cbil.upenn.edu/cgi-bin/tess/tess), and was highlighted in red. **B**. Binding of WT1 to WT1 binding site in AKT gene promoter in vivo by ChIP assay. Genomic DNA and input chromatin (input), which represent portions of sonicated chromatin before immunoprecipitation, were both used as positive controls.

WT1 (−Ex5/+KTS) binds to a novel recognition sequence which has been defined as (5′-GGAGG(A/G)-3′) [28]. In order to further confirm that the WT1 regulate AKT expression directly, we predicted the putative promoter region of AKT using the promoter scan website (http://www-bimas.cit.nih.gov/molbio/proscan), and predicted WT1 binding motif in AKT promoter using the Promoter and Transcription Factor Analysis web site (http://www.cbil.upenn.edu/cgi-bin/tess/tess). There are 10 potential WT1 binding site located upstream of the transcriptional start site (Figure 5A). In order to confirm our hypothesis that WT1 (−Ex5/+KTS) directly binds to the AKT promoter, we performed Chip assays in A549 cells. As shown in Figure 5B, fragment of the AKT promoter was immunoprecipitated from the A549 cell lysate using the anti-WT1 antibody, but not from control cell lysate. This indicated that there is a direct, physical interaction between WT1 and the AKT promoter, and demonstrated that AKT is a direct target of WT1 isoform C.

### Inhibition of WT1 expression increases DDP induced apoptosis in A549 lung cancer cells

**Figure 6:**
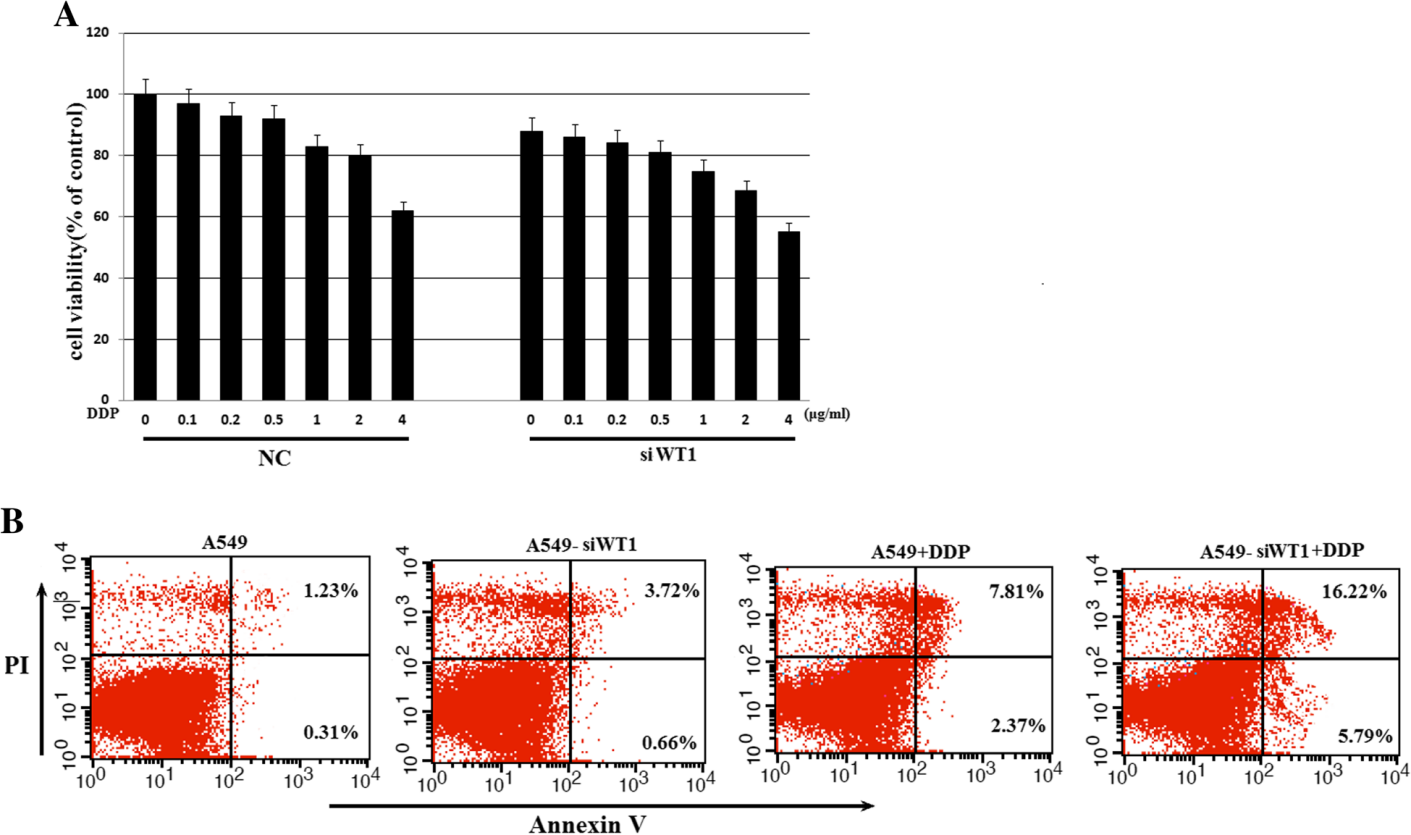
**Effects of WT1 siRNA in combination with DDP treatment on cell proliferation and apoptosis. A**. A549 cells were transfected with WT1 siRNA for 24 h, and then treated the cells with different concentrations of DDP for another 48 h, and cell viability was then analyzed by MTT assay. **B**. DDP-induced apoptosis in different A549 cells was assessed by Annexin V-PI staining. A549 cells were transfected with si-WT1 for 24 h, and then treated with or without 1 μg/ml of DDP for 24 h, cells were dual-stained with Annexin V and PI, apoptotic cells were then analyzed by flow cytometry. Three independent experiments were performed and all gave similar results. The percentage of apoptotic cells was calculated with WinMDI software.

We observed DDP treatment could reduce the WT1 expression effectively via the PI3K/AKT pathway, hence, we examined the effect of DDP treatment in combination with WT1 siRNA transfection. A549 cells were seeded into 6 well plates and cultivated until 80% confluency. Cells were firstly transfected with WT1 siRNA, 24 h post transfection, different concentrations of DDP were added for another 48 hours, and cell viability was then analyzed by MTT assay. In this experiment, the pre-transfection of si-WT1 before DDP exposure increased the sensitivity to DDP in lung cancer cells at all concentrations of DDP examined compared with NC pre-treated group (Figure 6A). DDP is known to exert its cytotoxic effect through induction of apoptosis, and hence, we investigated whether the increased DDP sensitivity observed in WT1 knockdown cells could be related to effects on apoptosis. The extent of apoptosis was investigated by FACS assay using the AnnexinV/PI staining kit. (Figure 6B) showed that the WT1 knockdown cells were more sensitive to DDP-induced apoptosis than the control cells. Taken together, these results clearly show that the silencing of the WT1 protein by WT1 siRNA makes the cells more prone to DDP-induced apoptosis.

## Discussion

Lung cancer is the first most common cause of cancer-related death in the world with very limited therapeutic options. Thus, new therapeutic strategies are urgently needed. WT1 has been identified as a potential molecular target for leukemia and some solid tumors, studies also demonstrated a correlation between WT1 expression and overall patient survival. There was also a study showed that WT1 is over expressed in lung cancer, although in their report, the authors observed a correlation between WT1 expression and patient survival, there was no data showing the implication of WT1 in lung cancer biology.

In the present study, real-time RT-PCR revealed 100% of WT1 positive cases of lung cancer, 56% of them express high levels of WT1, and A549 lung cancer cells also express high levels of WT1. Our result also shown that 89% of the tissue samples and the A549 cell line stably express the isoform C of WT1 gene without mutations/deletion. The WT1 targeted siRNA transfection induced the cell cycle arrested at the G_1_ phase through induction of p21 expression in WT1^+^ A549 cell lines but has little effect in WT1^-^PC14 lung cancer cell line; although we can’t detected the apoptotic cells by flow cytometry 24 h post WT1 siRNA transfection alone, which could due to the targeted siRNA we designed was targeting the Exon 8; we actually found that in A549 cells, the WT1 inhibition increased the expssion of proapoptotic protein BAX, BAK, and decreased the antiapoptotic protein Bcl-XL, Mcl-1 expression.

DDP is a standard chemotherapy reagent for treating lung cancer, our result indicated that DDP is able to inhibit WT1 expression in A549 cells in a dose- and time-dependent manner. After incubation with 1 or 2 μg/ml of DDP, the expression of WT1 mRNA and WT1 protein level was significantly down regulated mainly at the higher concentration. As many studies demonstrated that over activated PI3K/AKT pathway activity may lead to DDP resistance in malignant cells [29,30], inhibition of this signaling pathway increased the sensitivity of cancer cells to DDP treatment [29-33], and combined treatment of PI3K inhibitors with DDP also got promising results [34]. We thus considered that whether DDP treatment has any effect on PI3K/AKT pathway activity in A549 lung cancer cells. So we detected the phospholated form of AKT post DDP treatment. As expected, DDP treatment also decreased the expression of p-AKT, a study also pointed out that WT1 is the downstream target of AKT [17], we thus then hypothesized that DDP could reduce the WT1 expression via the PI3K/AKT pathway. As we expected, the PI3K pathway inhibitor treatment decreased the WT1 expression as well, furthermore, transfection of AKT targeted siRNA reduced the WT1 expression by nearly 50%. Interestingly, we found that when we knocking down the WT1 by siRNA transfection, the p-AKT was also reduced, though not as much as AKT targeted siRNA did, forced overexpression of WT1 in 293 T cells increased the p-AKT as well. As WT1 was a transcription factor, we are wondering if WT1 regulate the AKT expression through promoter binding, and the Chip assay showed that isoform C of WT1 interacts with the AKT promoter in vivo. So our results demonstrated that DDP reduced the WT1 expression through inhibition of the PI3K/AKT signal pathway, and knock down of WT1 could also lead to reduced AKT activity through decreased p-AKT level. So we concluded that there is a positive feedback loop between WT1 and the PI3K/AKT pathway activity.

All of the above lead us to hypothesize that down-regulation of WT1 gene expression maybe implicated a role in DDP-dependent inhibition of cell proliferation. We thus inhibited WT1 expression with siRNA prior to incubation with DDP, the result clearly showed that the down-regulation of WT1 with siRNA prior to DDP treatment resulted in a dramatic inhibition of cell proliferation even at low concentrations at 0.5 μg/ml of DDP. Moreover, WT1 siRNA increased DDP induced apoptosis in A549 lung cancer cells. So, WT1 may serve as a marker for DDP sensitivity in WT1^+^ lung cancer cell lines. The experiment data shown by the group of Glienke [20] also predicted the increased the sensitivity of curcumin by the ability to inhibit WT1 gene expression.

In conclusion, our study demonstrated that isoform C of WT1 is conservely expressed in lung cancer patients and indicates that the down-regulation of WT1 mediated by siRNA could inhibit the growth of lung cancer cells effectively, arrested the cell cycle at the G_1_ phase, and may enhance the lung cancer cell sensitivity to DDP treatment via the PI3K/AKT signaling pathway. We consider that siRNA against WT1 in combination with DDP treatment might be of potential value for the treatment of human lung cancer, and the associated experiment were undergo.

## Competing interests

On the behalf of the authors I indicate that none of the authors have any financial disclose or any personal relationships with other people or organizations that could inappropriately influence (bias) this work.

## Authors' contributions

XW collected the tissue samples and classified the NSCLC stages; PG carried out the molecular genetic studies, participated in the sequence alignment; ML prepared Total RNA and performed the RT-PCR assay; FL did the promoter analysis and CHIP assay; YYW and YROY performed the real-time PCR and analyzed the data; LL did all of the cell cuture and transfection studies; JXW carried out MTT studies; XC carried out the Western Blot analysis; TH carried out the cell cycle and apoptosis analysis; HHZ drafted the manuscript; KD carried out the statistic analysis, and made changes. All authors read and approved the final manuscript.
